# Dynamic wheelchair seating positions impact cardiovascular function after spinal cord injury

**DOI:** 10.1371/journal.pone.0180195

**Published:** 2017-06-30

**Authors:** Jessica A. Inskip, Henrike (Rianne) J. C. Ravensbergen, Inderjeet S. Sahota, Christine Zawadzki, Lowell T. McPhail, Jaimie F. Borisoff, Victoria E. Claydon

**Affiliations:** 1 Department of Biomedical Physiology and Kinesiology, Simon Fraser University, Burnaby, British Columbia, Canada; 2 International Collaboration on Repair Discoveries (ICORD), University of British Columbia, Vancouver, British Columbia, Canada; 3 British Columbia Institute of Technology (BCIT), Burnaby, British Columbia, Canada; University of Illinois at Urbana-Champaign, UNITED STATES

## Abstract

**Background:**

Innovative wheelchairs allow individuals to change position easily for comfort and social situations. While these wheelchairs are beneficial in multiple ways, the effects of position changes on blood pressure might exacerbate hypotension and cerebral hypoperfusion, particularly in those with spinal cord injury (SCI) who can have injury to autonomic nerves that regulate cardiovascular control. Conversely, cardiovascular benefits may be obtained with lowered seating. Here we investigate the effect of moderate changes in wheelchair position on orthostatic cardiovascular and cerebrovascular reflex control.

**Methods:**

Nineteen individuals with SCI and ten neurologically-intact controls were tested in supine and seated positions (neutral, lowered, and elevated) in the Elevation^™^ wheelchair. Participants with SCI were stratified into two groups by the severity of injury to cardiovascular autonomic pathways. Beat-to-beat blood pressure, heart rate and middle cerebral artery blood flow velocity (MCAv) were recorded non-invasively.

**Results:**

Supine blood pressure and MCAv were reduced in individuals with lesions to autonomic pathways, and declined further with standard seating compared to those with preserved autonomic control. Movement to the elevated position triggered pronounced blood pressure and MCAv falls in those with autonomic lesions, with minimum values significantly reduced compared to the seated and lowered positions. The cumulative duration spent below supine blood pressure was greatest in this group. Lowered seating bolstered blood pressure in those with lesions to autonomic pathways.

**Conclusions:**

Integrity of the autonomic nervous system is an important variable that affects cardiovascular responses to orthostatic stress and should be considered when individuals with SCI or autonomic dysfunction are selecting wheelchairs.

**Sponsorship:**

This work was supported in part by the Heart and Stroke Foundation of British Columbia and the Yukon (V.E.C).

## Introduction

Innovative variable positioning wheelchairs facilitate mobility and improve quality of life for wheelchair users. Some of these new devices permit dynamic modification of seating position, height, and tilt, to better navigate physical and social barriers. Seating positions can easily be adjusted in real time to suit particular activities, maximizing function, efficiency and independence [1, 2]. There may also be physiological benefits to dynamic seating, including reduction of pain, improved comfort, relieving pressure points, increasing bone density and improving baroreflex function [3–11]. However, altered seating positions likely also influence the physiological stressors placed on the cardiovascular system.

More upright seating positions challenge blood pressure regulation due to increases in orthostatic stress, which cause large fluid shifts to the lower body and abdomen, thus reducing venous return to the heart. This would normally be mitigated by autonomically-mediated baroreflex responses that increase heart rate and contractility, and increase sympathetic vasoconstriction. However, if these reflexes are impaired then this compensation can be insufficient to maintain blood pressure [12]. Indeed, impaired blood pressure regulation is a concern for individuals with high-level (above T5) spinal cord injury (SCI) with damage to autonomic (sympathetic) pathways that regulate the heart and vasculature [13]. In particular, sympathetic control of the vascular resistance and capacitance regions in the splanchnic circulation is critical to regulate blood pressure [14, 15]. Damage to cardiovascular sympathetic pathways results in low resting blood pressure and further hypotension can be triggered by positional changes [16–18]. A drop in blood pressure of ≥20 mmHg systolic and/or ≥10 mmHg diastolic constitutes orthostatic hypotension (OH) [19, 20]. OH is associated with low cerebral blood flow, and this likely contributes to the chronic fatigue experienced by many individuals with SCI [21–24]. Symptoms of OH limit individuals with SCI using standing frames and doing other physiotherapy exercises [25, 26]. A lowered wheelchair position, conversely, which compresses the abdominal area, could benefit venous return from the key vascular resistance and capacitance regions in the splanchnic circulation—similar to the effect of using an abdominal binder [27–29]. This might improve blood pressure and cardiac output [30].

**Fig 1:**
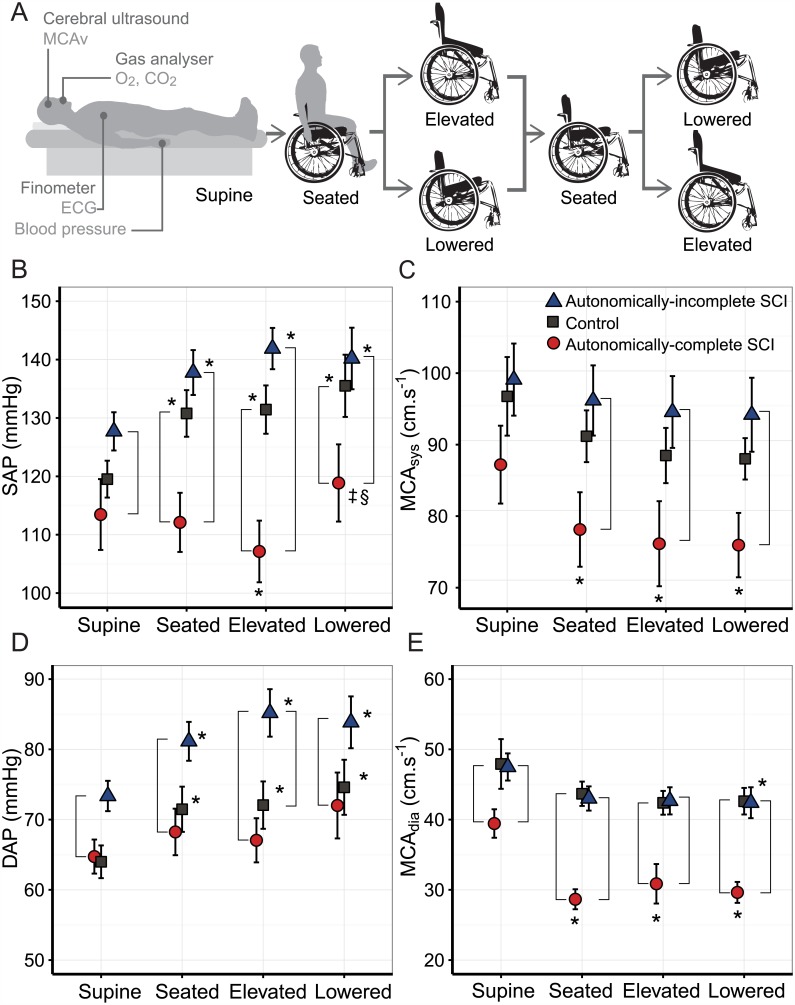
Blood pressure and cerebral blood flow responses to different wheelchair seating positions. A. Experimental protocol outline of crossover design: individuals were randomly assigned to move from the standard seating position to the maximally elevated or lowered position, and then returned to the standard seating position, followed by the opposite position (each block lasting 15 minutes). Throughout testing we continuously recorded blood pressure and electrocardiogram (ECG) waveforms using the Finometer device; end tidal oxygen (O_2_) and carbon dioxide (CO_2_) levels using a gas analyser; and middle cerebral artery blood flow velocity (MCAv) using cerebral ultrasound. B-E. Grouped mean data (± SEM) are presented in the supine, seated, elevated, and lowered wheelchair seating positions. Data were averaged over the last five minutes of each 15-minute trial. B. Systolic arterial pressure (SAP); C. systolic blood flow in the middle cerebral artery (MCA_sys_); D. diastolic arterial pressure (DAP); and E. diastolic blood flow in the middle cerebral artery (MCA_dia_) are presented. Vertical adjoining lines denote significant differences between indicated groups; asterisk (*) indicates significant difference from supine position; double dagger (‡) indicates significant difference from seated position; double S (§)indicates significant difference from elevated position.

Here we tested the effect of wheelchair seating position on cardiovascular and cerebrovascular function using a variable positioning manual wheelchair. We aimed to determine the effects of elevated, standard and lowered wheelchair seating positions on blood pressure, heart rate and cerebral blood flow (Fig 1A). Responses were compared between individuals with SCI who have cardiovascular autonomic dysfunction (autonomically-complete SCI), those with SCI but no damage to cardiovascular autonomic pathways (autonomically-incomplete SCI), and able-bodied controls. We hypothesized that elevated seating positions would exacerbate the orthostatic impairment in individuals with damage to cardiovascular autonomic pathways, but not in neurologically-intact controls or those with autonomically-incomplete SCI. We also hypothesized that lowered seating positions would ameliorate this effect.

## Materials and methods

We certify that all applicable institutional and governmental regulations concerning the ethical use of human volunteers were followed during the course of this research. This study conformed to the principles outlined in the Declaration of Helsinki [31], and received ethical approval from the Simon Fraser University Office of Research Ethics, and the Vancouver Coastal Health Research Institute. All participants provided written informed consent.

### Participants

All participants were ≥18 years old, apparently healthy and free of hypertension, diabetes mellitus and other overt cardiovascular disease. Nineteen individuals with SCI (8 women) who were regular wheelchair-users for at least one year participated. All participants with SCI and ten healthy able-bodied controls (4 women) were studied in elevated, lowered, and standard seating positions (Elevation^™^ wheelchair, PDG Mobility Technologies, Vancouver, BC) [1].

Neurological classification of level and severity of SCI was determined from the American Spinal Injury Association Impairment Scale (AIS) [32]. Participants with SCI were subdivided into two groups (autonomically-incomplete SCI and autonomically-complete SCI) based on the presence or absence of cardiovascular autonomic dysfunction. We considered an individual to have severe injury to spinal cardiovascular autonomic pathways, and therefore an autonomically-complete lesion, if they had: a) a lesion above T5; *and* b) low supine plasma noradrenaline spillover (<0.5 nmol/L); *and* c) low systolic blood pressure variability in the low frequency range (~0.1 Hz, Power < 2 mmHg^2^), as is standard in our laboratory [18, 33, 34].

### Equipment

Beat-to-beat blood pressure and lead II electrocardiogram (ECG) were recorded continuously (Finometer Pro, Finapres Medical Systems BV, Amsterdam, Netherlands). Middle cerebral artery (MCA) blood flow velocity was measured bilaterally using transcranial Doppler ultrasound with 2 MHz probes through the temporal windows (Doppler Box, Compumedics DWL, Singen, Germany) attached to a headband to maintain constant angles of insonation. Partial pressures of expired oxygen and carbon dioxide (P_ET_CO_2_) were recorded on a breath-by-breath basis (O_2_Cap, Oxigraf Inc, Mountain View, CA). All recordings were sampled at 1 KHz (Powerlab 16/30, AD Instruments, Colorado Springs, CO), acquired using LabChart (AD Instruments), and stored for offline analysis.

### Experimental protocol

Participants were asked to abstain from caffeine or alcohol and avoid strenuous exercise for 12 hours before testing. Testing was carried out in a quiet room at 20°C. Participants were instrumented while lying supine, and all parameters were recorded for 15 minutes. Participants then transferred to the Elevation^**™**^ wheelchair (back height 30 cm) in the neutral seated position (horizontal seat, 90° shin-to-seat angle, 90° seat-to-back angle) for 15 minutes. In a crossover design, individuals were randomly assigned to move to the maximally elevated (seat back raised, 120° shin-to-seat angle, 115° seat-to-back angle), or lowered position (seat back lowered, 75° shin-to-seat angle, 85° seat-to-back angle), and then returned to the neutral seated position, followed by the opposite position, with each block lasting 15 minutes (Fig 1A). End points for testing were determined *a priori*, and included any of: systolic arterial blood pressure below 80 mmHg; symptoms of presyncope, such as dizziness; or participant request.

### Data analyses

We applied a low-pass filter (<50 Hz) to remove electrical noise from the ECG signal and a median filter (~51 samples, or 0.05 s) to remove artifacts from the MCA signal. Beat-to-beat heart rate (HR), systolic (SAP), diastolic (DAP), mean (MAP) arterial pressure, as well as MCA systolic (MCA_sys_), diastolic (MCA_dia_), and mean (MCA_mean_) cerebral blood flow velocities, and breath-by-breath P_ET_CO_2_ were determined using cyclic peak detection measurement algorithms in LabChart. For each individual, the most reliable and consistent signal from either the left or right MCA was used for analysis (the side with the greatest number of beats with faithful envelope detection). Stroke volume (SV) and cardiac output (CO) were derived using the Modelflow^™^ technique [35]. In addition to reporting absolute values of cerebral blood flow velocity, we also normalised these data with respect to the supine baseline (percentage change) to control for any differences in initial probe position between participants.

A five-beat moving average was applied across all variables. Average values were calculated over the last five minutes of each condition (supine, seated, lowered, seated repeat, and elevated). Values from the two neutral seated conditions were averaged as they were not significantly different from each other.

In addition to the five minute averages, we computed three additional variables: minimum blood pressure (nadir), the timing of the nadir, and the overall orthostatic burden. The timing and size of the blood pressure nadir in response to orthostatic stress varies between individuals and together this information can provide insight into the severity of sympathetic dysfunction [36]. Nadir values were calculated from five-beat moving averages and the time at nadir determined. The *duration* of hypotension following postural change and ability to recover blood pressure during orthostasis have been identified as key measures of severity of OH [36, 37]. We quantified the overall orthostatic burden of each position as the cumulative magnitude and duration spent below resting supine SAP levels. The cumulative area under the curve (AUC) was calculated as the sum of the difference between supine SAP and SAP at each beat [37].

### Statistical analyses

Data processing was conducted using R Version 3.0.1 and data were analysed using JMP Version 10.0 and SigmaPlot Version 12. Descriptive statistics were calculated by group for the supine variables. Non-parametric data were log transformed prior to statistical analyses. SCI-specific measures such as duration of injury were compared using t-tests. Distribution of level and severity of injury between groups was compared using Fisher’s exact test. One-way ANOVAs were used for comparison of demographic information and supine variables, with Tukey’s post-hoc test to examine between group differences. Cardiovascular responses to different wheelchair positions were compared using a two-way ANOVA (participant subgroup and seat position) with repeated measures on one factor (position). The dependent variable was the absolute cardiovascular parameter. Where main effects were present, post-hoc comparisons were conducted using the Holm-Sidak method. Statistically significant differences were assumed where α<0.05.

## Results

Participant characteristics are shown in Table 1. We did not detect any differences in sex distribution, age, height, or weight between groups. For comparisons between the two SCI groups, we did not detect any differences in time since injury, but there were more participants with cervical injuries in the autonomically-incomplete SCI group (p = 0.002), and there tended to also be more participants with motor and sensory complete lesions (AIS A) in this group (p = 0.057).

**Table 1:** Participant demographics and baseline cardiovascular parameters.

		Control	Autonomically-incomplete SCI	Autonomically-complete SCI
**Sample size**		10	12	7
**Age (**years)		31.9 (8.3)	42.6 (10.7	37.0 (8.1)
**Sex** (male:female)		6:4	6:6	5:2
**Time since injury** (years)		-	18.9 (4.1)	16.6 (3.6)
**Lesion level**	Cervical	-	1	6*
	Thoracic	-	11	1*
**AIS grade**	A	-	8	1
	B/C/D	-	4	6
**Height** (cm)		174 (6)	170 (8)	176 (12)
**Weight** (kg)		73 (11)	67 (10)	68 (12)

Data are presented as group means with standard deviation shown in brackets. SCI groups were divided according to autonomic completeness of injury. Cardiovascular variables were recorded in the supine position for 15 minutes. Asterisk (*) indicates significant difference from autonomically-incomplete SCI (p<0.05). Abbreviations: AIS, American Spinal Injury Association Impairment Scale; SCI, spinal cord injury.

Supine cardiovascular and cerebrovascular recordings showed that the autonomically-complete SCI group had significantly lower SAP, DAP, MCA_dia_ and MCA_mean_ compared to the autonomically-incomplete group (Table 2, all p<0.05). MCA_dia_ was also significantly lower in the autonomically-complete SCI group compared to the controls (p<0.05). The autonomically-incomplete SCI group had higher DAP (p = 0.015) and MAP (p = 0.035) compared to the control group. The remaining supine cardiovascular and cerebrovascular parameters were not significantly different between groups.

**Table 2:** Mean cardiovascular variables in different wheelchair positions.

		Control		Autonomically-incomplete SCI		Autonomically- complete SCI	
		Raw	% supine	Raw	% supine	Raw	% supine
**SUPINE**	**SAP** (mmHg)	120 (10)		128 (11)		**113 (16)** ^I^	
	**DAP** (mmHg)	**64 (7)** ^I^		73 (7)		**65 (6)** ^I^	
	**MAP** (mmHg)	**71 (12)** ^I^		83 (9)		80 (8)	
	**MCA**_**sys**_ (cm.s^-1^)	96 (18)		99 (18)		87 (15)	
	**MCA**_**dia**_ (cm.s^-1^)	48 (11)		47 (7)		**39 (5)** ^C^^**,**^ ^I^	
	**MCA**_**mean**_ (cm.s^-1^)	66 (14)		67 (12)		**56 (9)** ^I^	
	**HR** (bpm)	65.1 (8.2)		67.0 (9.1)		63.8 (13.7)	
	**SV** (mL)	96.1 (12.6)		90.9 (14.8)		88.4 (12.8)	
	**CO** (L.min^-1^)	6.2 (0.9)		6.0 (1.1)		5.8 (1.3)	
**SEATED**	**SAP** (mmHg)	131 (13)*		138 (13)*		**112 (13)** ^C^^,^ ^I^	
	**DAP** (mmHg)	71 (10)*		81 (10)*		**68 (9)** ^I^	
	**MAP** (mmHg)	82 (16)*		93 (13)*		85 (7)	
	**MCA**_**sys**_ (cm.s^-1^)	91 (12)	96 (10)	96 (17)	97 (6)	77 (14)* ^I^	90 (9)*
	**MCA**_**dia**_ (cm.s^-1^)	44 (5)	95 (16)	43 (6)	91 (8)	29 (4)* ^C^^,^ ^I^	73 (10)* ^C^^,^ ^I^
	**MCA**_**mean**_(cm.s^-1^)	61 (8)	95 (14)	62 (11)	93 (7)	45 (8)* ^C^^,^ ^I^	81 (6)* ^C^^,^ ^I^
	**HR** (bpm)	71.1 (9.6)*		74.5 (10.4)*		70.7 (17.6)*^E^	
	**SV** (mL)	85.2 (11.2)*		74.8 (14.4)*		74.9 (10.0)* ^E^	
	**CO** (L.min^-1^)	6.1 (0.8)		5.6 (1.2)		5.0 (1.3)	
**ELEVATED**	**SAP** (mmHg)	131 (13)*		142 (12)*		107 (14)* ^C^^,^ ^I^	
	**DAP** (mmHg)	72 (11)*		85 (12)*^C^		**67 (8)** ^I^	
	**MAP** (mmHg)	81 (14)*		98 (16)* ^C^		**82 (7)** ^I^	
	**MCA**_**sys**_ (cm.s^-1^)	88 (13)	93 (8)	94 (18)	95 (5)	75 (16)* ^I^	87 (12)*
	**MCA**_**dia**_ (cm.s^-1^)	42 (5)	92 (14)	43 (7)	90 (9)	31 (7)* ^C^^,^ ^I^	79 (17)* ^C^^,^ ^I^
	**MCA**_**mean**_(cm.s^-1^)	58 (8)	91 (11)	61 (11)	91 (5)	45 (12)* ^C^^,^ ^I^	80 (13)* ^C^^,^ ^I^
	**HR** (bpm)	74.7 (12.1)*		76.6 (11.2)*		79.4 (17.3) * ^S^^,^ ^L^	
	**SV** (mL)	80.2 (13.0)*		69.3 (12.6)*		62.3 (12.0)* ^C^^,^ ^S^^,^ ^L^	
	**CO** (L.min^-1^)	5.9 (0.9)		5.3 (1.0)*		4.5 (0.8)* ^C^	
**LOWERED**	**SAP** (mmHg)	136 (17)*		140 (18)*		**119 (17)** ^S^^,^ ^E^^,^ ^C^^,^ ^I^	
	**DAP** (mmHg)	75 (12)*		84 (13)*		**72 (12)** ^I^	
	**MAP** (mmHg)	85 (18)*		96 (15)*		89 (4) *	
	**MCA**_**sys**_ (cm.s^-1^)	87 (9)	96 (13)	94 (18)	95 (8)	75 (12)* ^I^	87 (8)*
	**MCA**_**dia**_ (cm.s^-1^)	43 (6)	95 (18)	42 (8)	88 (8)	30 (4)* ^C^^,^ ^I^	76 (7)* ^C^^,^ ^I^
	**MCA**_**mean**_(cm.s^-1^)	59 (8)	95 (16)	61 (12)	91 (6)	45 (7)* ^C^^,^ ^I^	81 (7)* ^C^^,^ ^I^
	**HR** (bpm)	71.3 (10.3)*		75.9 (13.2)*		71.7 (13.6) * ^E^	
	**SV** (mL)	85.4 (11.2)*		69.3 (17.0)* ^C^		76.5 (10.4)* ^E^	
	**CO** (L.min^-1^)	6.0 (0.7)		5.2 (1.3)*		5.0 (0.5)	

The last five minutes of each position were averaged for each subject. Data for the two seated positions (90° shin-to-seat angle) were averaged. Data are presented as group means, with standard deviation in brackets. Both raw values and percentage of supine values are presented (% supine). Asterisk (*) indicates significantly different from supine position (p<0.05);

^**S**^ indicates significantly different from seated position;

^**L**^ indicates significantly different from lowered position;

^**E**^ indicates significantly different from elevated position;

^**C**^ indicates significantly different from able-bodied control group (p<0.05);

^**I**^ indicates significantly different from autonomically-incomplete SCI group (p<0.05).

Abbreviations: CO, cardiac output; DAP, diastolic arterial pressure; HR, heart rate; MAP, mean arterial pressure; MCA, middle cerebral artery; MCAdia, MCA diastolic blood flow; MCAsys, MCA systolic blood flow; MCAmean, MCA mean blood flow; SAP, systolic arterial pressure; SCI, spinal cord injury; SV, stroke volume.

Movement from a supine to seated position had different effects on SAP depending on autonomic completeness of injury (p = 0.0014, Fig 1B, Table 2), as we have previously reported [34, 38]. SAP increased in controls (p≤0.001), and in autonomically-incomplete SCI (p = 0.024), and failed to increase in the autonomically-complete SCI group. Movement from the seated to elevated position did not further affect SAP in autonomically-incomplete SCI and control groups. In the elevated position, mean SAP was lower in the autonomically-complete SCI group compared to resting supine levels (p = 0.037). Conversely, the lowered position increased SAP compared to both standard seating and elevated seating in the autonomically-complete SCI group (p = 0.029). Movement to the lowered position did not change SAP in autonomically-incomplete SCI and control groups. Compared to the other two groups, SAP was lower in the autonomically-complete SCI group during seated, elevated, and lowered positions (Fig 1, Table 2).

Similarly to SAP, DAP and MAP increased from supine to seated, elevated and lowered levels in control and autonomically-incomplete SCI groups (all p<0.05, Fig 1D, Table 2). There were no significant differences in DAP or MAP between positions in the autonomically-complete SCI group except for the MAP in the lowered position, which was increased compared to supine.

HR increased from supine values during seated, elevated and lowered positions in all three groups (all p<0.05). In the autonomically-complete SCI group only, the HR in the elevated position was higher than in the seated and lowered positions.

SV decreased in all groups from supine to all seated positions (Table 2). In the autonomically-complete group only, movement from the seated to elevated position further reduced SV (p<0.05). In the control group, the reduction in SV was compensated by HR increases, so CO was unchanged. However, both SCI groups showed a decrease in CO in the elevated position; in the autonomically-complete group CO during elevated seating was significantly lower than in controls (Table 2).

Group average cerebrovascular parameters in different wheelchair positions are also reported in Table 2. In all seating positions, MCA_sys_ was reduced compared to supine in those with autonomically-complete SCI (p = 0.02), but not in those with autonomically-incomplete SCI or controls (Fig 1C). In all seated positions, MCA_sys_ was significantly lower in the autonomically-complete SCI group compared to the autonomically-incomplete group (p<0.05).

Diastolic cerebral blood flow velocity (MCA_dia_) was particularly affected by orthostatic challenge in the autonomically-complete SCI group, similar to our previous report [34] (Fig 1E). MCA_dia_ was significantly lower in the autonomically-complete SCI group compared to both groups in the seated, elevated, and lowered positions (all p<0.05). Movement from the seated to the elevated position did not further decrease MCA_dia_ in the autonomically-complete SCI group. Similar group results and changes with position were seen in MCA_mean_. Significant changes in P_ET_CO_2_ were not detected and do not explain these results.

**Fig 2:**
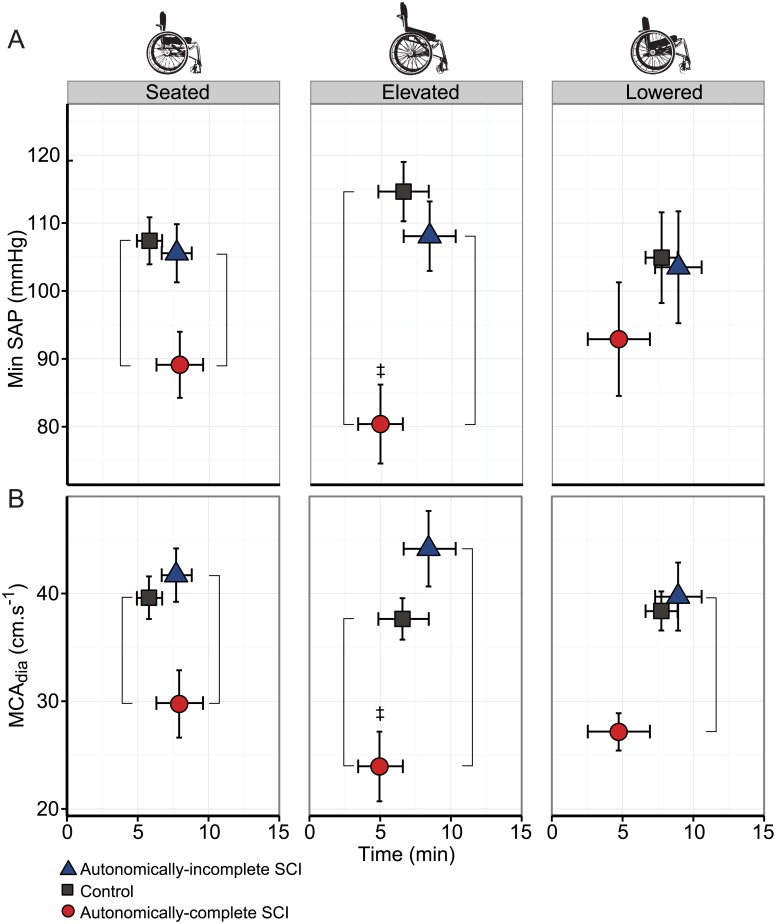
Nadir systolic arterial pressure and cerebral blood flow in seated, elevated and lowered wheelchair positions. A. Nadir systolic arterial pressure (SAP) and time at nadir in each wheelchair position. B. Diastolic middle cerebral artery blood flow (MCA_dia_) at nadir systolic arterial pressure. Vertical adjoining lines denote significant differences between indicated groups; double dagger (‡) indicates significant difference from seated position.

In the seated and elevated positions, nadir SAP was significantly lower in the autonomically-complete SCI group compared to both autonomically-incomplete (p<0.05, Fig 2) and control groups (p<0.05). There were no significant differences in minimum SAP in the lowered position between the three groups (p>0.05). Within the autonomically-complete SCI group, minimum SAP was significantly lower in the elevated position compared to seated (p<0.05). In all positions, there was no statistical difference in the timing of the SAP nadir between groups over the 15-minute period (all p>0.05). The nadir MCA_dia_ was lower in the autonomically-complete SCI group compared to both groups in all wheelchair positions (all p<0.05, Fig 2). Considering the change from seated to elevated, MCA_dia_ was significantly lower in the autonomically-complete SCI group (p = 0.039).

**Fig 3:**
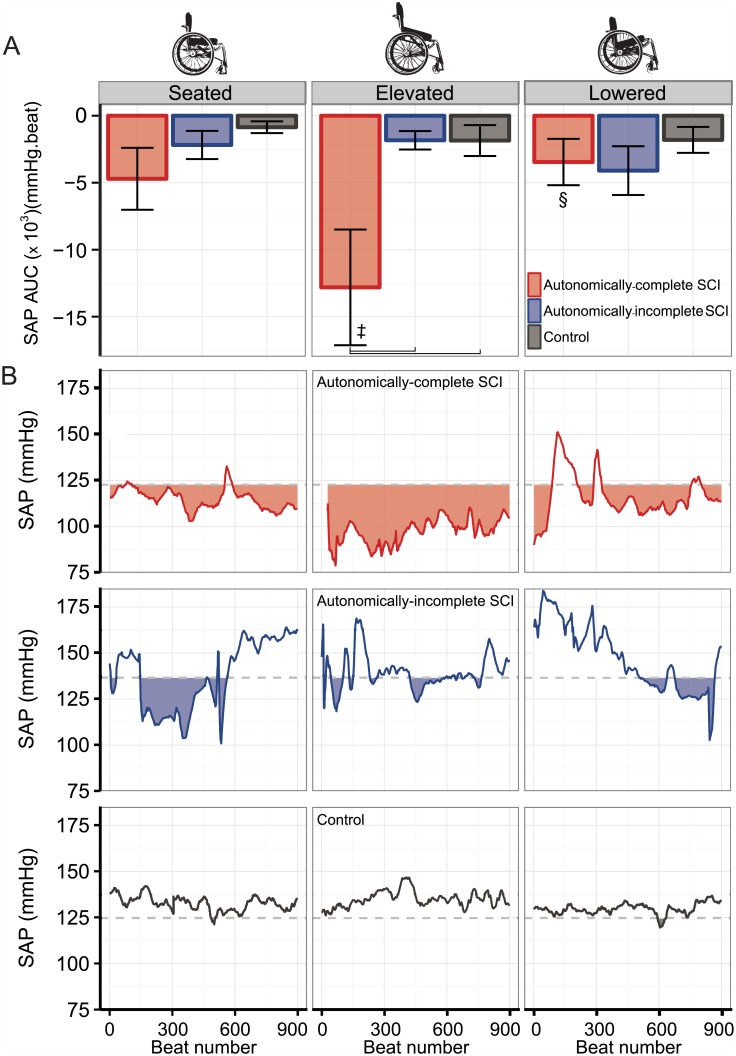
Cumulative orthostatic burden in seated and elevated wheelchair positions. A. Cumulative orthostatic burden was calculated as the cumulative area under the curve (AUC) for the duration of each wheelchair position: the difference between baseline systolic arterial pressure (SAP) and SAP multiplied by the duration of each beat. B. Example traces from a representative individual in each group. Dotted horizontal line indicates supine SAP for that individual and shaded area indicates regions below supine SAP. Vertical adjoining lines denote significant differences between indicated groups; double dagger (‡) indicates significant difference from seated position; double S (§) indicates significant difference from elevated position.

The cumulative orthostatic burden in each position is shown in Fig 3. There was no significant difference in AUC between groups in the seated position (p>0.05). However, the AUC of individuals with autonomically-complete SCI was greater in the elevated position compared to both seated and lowered positions, and was significantly different from autonomically-incomplete SCI and control groups (all p<0.05). There were no significant differences in orthostatic burden between the seated, elevated, and lowered positions in either the control or autonomically-incomplete SCI groups. Fig 3B shows an example of the OH burden in each condition from one representative individual in each group.

## Discussion

Wheelchair technologies continue to improve to meet the needs of individuals living with disability. Our results show that caution is warranted before encouraging all individuals to adopt and use new wheelchairs without prior education and planning. Individuals with high-level SCI and damage to autonomic pathways tend to have low resting blood pressure, particularly when seated. In this population, moderate changes in body position can result in periods of low blood pressure and cerebral blood flow that can potentially trigger symptoms of presyncope or even syncope [18, 34]; lowered seating positions may somewhat mitigate these effects.

We showed that cardiovascular homeostasis in individuals with autonomically-complete SCI is perturbed by positional changes. Similar to our previous work [18, 34], averaged data showed lower seated blood pressure in this group compared to individuals with autonomically-incomplete SCI and controls. Movement to an elevated wheelchair position further challenged blood pressure, triggering pronounced falls in SAP and cerebral perfusion, with nadir values significantly lower than in the seated or lowered positions. There may be some concern about the cumulative burden of hypotension—and potentially cerebral hypoperfusion—over long periods in the elevated position. Hypotension in individuals with SCI has been associated with deficits in memory and attention processing speed [39, 40], as well as increased risk of cardiovascular disease and other secondary complications of SCI [41].

We found that lowered seating improved SAP, HR and SV compared to elevated seating in those with autonomically-complete SCI (Table 2) and could, therefore, be used as a counter-maneuver for individuals to bolster blood pressure. In fact, a lowered position is often the preferred seating position for individuals with high-level SCI because it improves trunk stability [1, 42]. These results highlight the importance of regularly returning to lowered seating positions for individuals using elevated seating options to limit overall time spent with low resting blood pressure. Dynamic wheelchairs facilitate frequent position changes that can help with blood pressure control and hopefully limit orthostatic symptoms and fatigue. While there was an improvement in SAP in the lowered position, this was not the case for MCA_sys_. This failure to improve cerebral perfusion despite improvements in blood pressure could be an indicator of impaired autoregulation, as we have previously reported in this population [34].

The benefit of the lowered seating position may be more apparent during dynamic exercise. However, we focused on static positioning and did not test cardiovascular function while wheeling. Body movement and dynamics during wheeling may accentuate the differences in cardiovascular parameters between positions. For example, the lowered position provides improved ergonomics for wheeling compared to the seated or elevated position, and individuals often lean forward to move the center of gravity forward [43]. This would further compress the abdomen and increase venous return to the heart [44]. Certainly, the increases in stroke volume, and blood pressure, and decreases in heart rate, that we observed in the lowered seating position would likely be beneficial during exercise, particularly in a population known to have difficulty mounting an appropriate cardiovascular response to exercise [16]. Therefore, the functional cardiovascular benefits of the lowered position might be enhanced during exercise; future work should evaluate the cardiovascular impact of lowered seating positions during exercise.

While not the main focus of this study, our results reiterate the dissociation between AIS Score, which classifies motor and sensory injury impairment, and the *autonomic* completeness of injury [18, 33, 34]. It is not always possible to predict autonomic injury from the status of motor and sensory pathways.

Given the disruption of sympathetic nerve pathways in those with autonomically-complete injuries, we might have expected greater impairments in cardiovascular control during orthostatic stress in this group; however, compensatory physiological adaptations, such as changes in the renin-aldosterone-angiotensin system [45] and antidiuretic hormone release [46], may occur over time. The extent of spasticity may also play an important role in these adaptations—muscle contractions associated with lower extremity spasticity can activate the skeletal muscle pump and improve venous return [47]. Conversely, muscle atrophy and flaccid paralysis might minimize lower limb blood flow and limit venous pooling and subsequent capillary filtration [48, 49]. Indeed, reduced arterial diameter and blood flow in the distal limbs has been noted in those with SCI [50], and might actually mitigate orthostatic intolerance. We did not evaluate the contribution of lower limb spasticity to cardiovascular control, but its role as an explanatory variable is of interest for future studies.

Here we considered only the acute impact of dynamic seating and associated orthostatic stress on cardiovascular control. However, there may be a training effect of repeated exposure to orthostatic stress [51]. This could explain, in part, the modest reduction in incidence and severity of OH with time after injury [52]. Whether this reflects a true reduction in the orthostatic burden through improved cardiovascular reflex control, increased tolerance to the symptoms of OH, or optimized treatment and management of OH is unclear. If orthostatic training is possible in individuals with SCI exposed to repeated orthostatic stresses, the potential concerns associated with dynamic seating might be mitigated over time, and could be considered a form of conditioning to improve cardiovascular responses [11].

We employed well-used techniques to assess cardiovascular function. However, the assessment of cerebral blood flow from indirect measurements of velocity using Doppler ultrasound relies on the assumption that the insonated vessel diameter remains constant [53]. Although we did not measure MCA diameter, it is thought to remain fairly constant [53]–except with large changes in P_ET_CO_2_ [54, 55], which did not occur in our study. The absolute values of MCA blood flow velocity can also be affected by angle of insonation and probe positioning. We minimized the effect of changes in probe positioning and angle of insonation within subjects during testing by clamping the probe in place with a headband. Because of the anatomical position of the vessel, the angle of insonation with respect to the middle cerebral artery is close to zero, so any small differences in the insonation angle between participants would have little effect on the resulting Doppler shift, and hence on the velocities recorded. Nevertheless, because of the theoretical impact of the angle of insonation on the absolute values of MCA blood flow velocity, we report both absolute values and percentage changes in MCA parameters (Table 2), with qualitatively similar results.

One limitation of this study is the smaller number of participants with cervical SCI—who comprise about half of all individuals living with SCI [56]. Given that individuals with high injury levels are most likely to experience abnormal blood pressure regulation, the current results may *underestimate* the severity of cardiovascular compromise. It is possible that this reflects self-selection of these individuals, because of known orthostatic intolerance or difficulty completing the transfers inherent in this study. In practice, it is likely that many individuals with high-level SCI would not elect to use wheelchairs with dynamic seating due to trunk instability; for those who do, cardiovascular complications should be taken into consideration.

Several new *powered* mobility alternatives have recently been developed for individuals with high-level SCI that pose similar challenges to cardiovascular control. These power wheelchairs permit a wide range of position flexibility, spanning from supine to standing [57, 58], enabling even individuals with very high-level SCI to access the benefits of standing. However, it may also make them vulnerable to severe orthostatic decreases in blood pressure. It would be interesting to investigate the effects of these wheelchairs on blood pressure and cerebral blood flow in individuals with high-level SCI—who are both the target user group and at the highest risk for cardiovascular dysfunction. Furthermore, exoskeletons have recently been approved by the FDA [59] and will soon be viable alternatives for individuals with mobility impairments [60]. Devices that incorporate lower limb locomotion, either partially-assistive or passive (e.g. using an exoskeleton), may bolster orthostatic blood pressures through lower limb skeletal muscle pumping activity [27]. Studies on these new devices and their impact on cardiovascular control will be critical to ensure appropriate design configurations that mitigate the orthostatic cardiovascular deficit.

The integrity of the autonomic nervous system is an important variable that affects cardiovascular responses to orthostatic stress and should be considered when individuals are selecting and configuring wheelchairs with dynamic seating options. These results have implications for individuals with SCI and also non-SCI wheelchair users with cardiovascular autonomic impairment such as individuals with multiple sclerosis, diabetic neuropathy, and autonomic failure syndromes. We hope this research will encourage clients, physicians and seating specialists to consider cardiovascular stressors when they are selecting possible wheelchairs. Discussion and education around identification of early symptoms of OH and presyncope should be included when individuals are making wheelchair decisions. The ability to make rapid position changes to recover blood pressure if individuals begin to feel symptoms of presyncope is an important consideration for wheelchair users—especially for those with severe autonomic impairment. With modest education and key contingency procedures in place, all wheelchair users should be able to access the myriad benefits of dynamic position changes to their health, independence, and quality of life.
